# Microstructure and Hydrogen Permeability of Nb-Ni-Ti-Zr-Co High Entropy Alloys

**DOI:** 10.3390/membranes12111157

**Published:** 2022-11-17

**Authors:** Egor Kashkarov, Dmitriy Krotkevich, Maxim Koptsev, Sergei Ognev, Leonid Svyatkin, Nahum Travitzky, Andrey Lider

**Affiliations:** 1 School of Nuclear Science and Engineering, National Research Tomsk Polytechnic University, 634050 Tomsk, Russia; 2 Department of Materials Science, Glass and Ceramics, Friedrich-Alexander-Universität Erlangen-Nürnberg, 91054 Erlangen, Germany

**Keywords:** high-entropy alloys, membranes, microstructure, ab initio calculation, hydrogen permeability

## Abstract

Hydrogen separation membranes are one of the most promising technologies for hydrogen purification. The development of high-entropy alloys (HEAs) for hydrogen separation membranes is driven by a “cocktail effect” of elements with different hydrogen affinities to prevent hydride formation and retain high permeability due to the single-phase BCC structure. In this paper, equimolar and non-equimolar Nb-Ni-Ti-Zr-Co high entropy alloys were fabricated by arc melting. The microstructure and phase composition of the alloys were analyzed by scanning electron microscopy and X-ray diffraction, respectively. The hydrogen permeation experiments were performed at 300–500 °C and a hydrogen pressure of 4 bar. In order to estimate the effect of composition and lattice structure on hydrogen location and diffusivity in Nb-Ni-Ti-Zr-Co alloy, ab initio calculations of hydrogen binding energy were performed using virtual crystal approximation. It was found that Nb-enriched and near equimolar BCC phases were formed in Nb_20_Ni_20_Ti_20_Zr_20_Co_20_ HEA while Nb-enriched BCC and B2-Ni(Ti, Zr) were formed in Nb_40_Ni_25_Ti_18_Zr_12_Co_5_ alloy. Hydrogen permeability tests showed that Nb_20_Ni_20_Ti_20_Zr_20_Co_20_ HEA shows lower activation energy and higher permeability at lower temperatures as well as higher resistance to hydrogen embrittlement compared to Nb_40_Ni_25_Ti_18_Zr_12_Co_5_ alloy. The effect of composition, microstructure and hydrogen binding energies on permeability of the fabricated alloys was discussed.

## 1. Introduction

Membrane technology is one of the most efficient approaches for hydrogen separation and purification due to low operating costs, high productivity and low power consumption [1,2]. Metal membranes have the highest selectivity for hydrogen separation. Thin Pd foils have a separation factor >1000 [1]. Palladium-based (Pd-based) membranes with a face centered cubic (fcc) lattice structure have the ability to dissociate and dissolve molecular hydrogen and thus exhibit excellent H_2_ permeability properties [3]. However, the high cost of Pd motivates researchers to develop composite membranes or alternative effective membrane materials [4].

The V metals group (V, Nb, Ta, etc.), with a more open body-centered cubic (BCC) lattice, are more permeable to hydrogen and are promising candidate materials for hydrogen separation membranes [5,6]. However, in most cases the single-element membranes of the V group suffer from hydrogen embrittlement (HE) and gas poisoning at high temperature, which does not allow their wide use for hydrogen separation in industry [7]. Thus, multicomponent alloys based on V group elements with a BCC structure, which are more resistant to embrittlement, are being developed [8,9]. It is well known that the composition of alloys significantly affects its hydrogen permeability and resistance to HE [10]. It was shown that alloying of Nb-based alloys with Ti and Ni can lead to a reduction of hydrogen embrittlement and permeability [11,12]. In addition to the formation of BCC phases, interdendritic TiNi compounds inhibit hydride formation. Ni stabilizes the alloy with reduced H absorption. The presence of Ti in Nb-based alloys increases the thermal stability and hydrogen absorption [9,13]. The addition of Zr in Ni-Nb-Zr-Co alloy increases hydrogen sorption and increases permeability; however, it reduces the resistance to HE. The addition of Co is effective in suppressing hydrogen embrittlement of Ni-Nb-Zr-Co alloy [14]. Tang et al. investigated multicomponent Nb_40_Ti_18_Zr_12_Ni_25_M_5_ (M = Al, Co, Cu, Pd) and showed that the Nb_40_Ti_18_Zr_12_Ni_25_Co_5_ alloy had the highest permeability of 3.8 × 10^−8^ (${\mathrm{molH}}_{2}m^{-1}s^{-1}{\mathrm{Pa}}^{-0.5}$), higher than that for Pd_0.23_Ag_0.77_ alloy, with a permeability of 1.6 × 10^−8^ (${\mathrm{molH}}_{2}m^{-1}s^{-1}{\mathrm{Pa}}^{-0.5}$), while maintaining stable performance [15].

Multicomponent alloys with more than four elements in near equimolar composition, also known as high-entropy alloys (HEAs), can be considered as membrane materials. The “cocktail effect” [16] of elements with different hydrogen affinities can provide low hydrogen embrittlement and high permeability due to the single phase BCC crystal structure. It was reported that high-entropy alloys have high mechanical strength, which is maintained over a wide temperature range, phase stability and low degradation rate [17]. Han et al. demonstrated high phase stability and mechanical properties for Nb_20_Ni_20_Ti_20_Co_20_Zr_20_ under compressive tests up to 800 °C [18]. However, there are no experimental works on the hydrogen permeability of this HEA. Therefore, in the present work, based on the positive effect of alloying elements we selected the Nb-Ni-Ti-Zr-Co system to fabricate equimolar and non-equimolar high-entropy alloys and investigated its microstructure and hydrogen permeability.

## 2. Materials and Methods

### 2.1. Material Preparation

Metal powders of Nb, Ni, Ti, Zr, Co (>99.5% purity) were mixed in a ball mill (AGO-2: Novits, Novosibirsk, Russia) to obtain homogeneous distribution of elemental powders. The powders were mixed with the rotation speed of 170 rpm for 1 h. Two series of powders with equimolar composition (Nb_20_Ni_20_Ti_20_Zr_20_Co_20_) and non-equimolar composition (Nb_40_Ni_25_Ti_18_Zr_12_Co_5_) were prepared. The mixed powders were pressed by a cold static uniaxial method at the pressure of 90 MPa in a closed mold. The alloy samples were fabricated by arc melting of the pressed powders on a water-cooled copper crucible in an argon atmosphere. Then, the samples were annealed in vacuum at 800 °C for 10 h. The resulting ingots were cut into disks of 10 mm in diameter and 0.7 mm in thickness. Then, the samples were ground using SiC paper with a grain size up to 5 µm. Finally, the samples were annealed in vacuum at 800 °C for 6 h. 

### 2.2. Characterization

The phase composition and crystalline structure of the fabricated alloys were analyzed by X-ray diffraction (XRD) using an XRD-7000S diffractometer (Shimadzu, Kyoto, Japan) with Cu*K*_α_ radiation (1.5410 Å wavelength) at 40 kV and 30 mA in the Bragg-Brentano geometry. The phases were identified using the PDF4+ 2021 database and SIeve software (ICDD, Newtown, PA, USA). The phase composition and lattice parameters were calculated by Rietveld refinement. The microstructure and elemental composition of the samples were analyzed by scanning electron microscopy (SEM) using Vega 3 (Tescan, Brno, Czech Republic) equipped with an energy dispersive X-ray spectroscopy (EDX) attachment.

### 2.3. Hydrogen Permeation Tests

**Figure 1:**
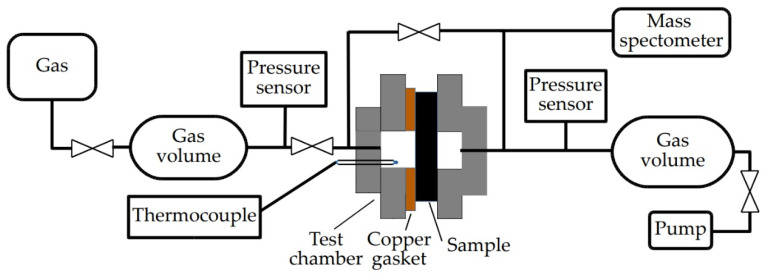
Schematic diagram of the test cell for hydrogen permeation experiments.

For the hydrogen permeation test, the samples were placed in the test cell with a copper seal gasket (Figure 1). The surface of the samples was covered by thin Pd film using magnetron sputtering. The active surface area of the membrane samples was 1.9 × 10^−5^ m^2^. Leak testing was carried out before and after the experiment. Hydrogen was supplied from one side of the membrane at room temperature. From the side of the gas outlet, the hydrogen signal was analyzed by a mass spectrometer. The experiment was started after the leakage test was valid. The chamber was evacuated to the residual pressure of 10^−3^ Pa from both sides of the sample. The sample was heated to 300–500 °C with a heating rate of 6 °C/min. After reaching the heating temperature, hydrogen gas (4 bar) was supplied to the one side of the sample.

The concentration of hydrogen permeated through the sample was measured by the change in pressure in known volumes:

(1) $$n\left(mol\right)=\frac{P\times V}{R\times T},$$

where *P*—pressure (Pa), *V*—volume (m^3^), *R*—gas constant, *T*—temperature (K).

The flux (*Φ*) was calculated from the difference in the amount of gas passed through the area of the sample in a period of time:

(2) $$\Phi =\frac{n1-n2}{t\times S},$$

where *n*1—initial concentration of gas (mol), *n*2—final concentration of gas (mol), *t*—time (s), *S*—active surface area of the sample (m^2^).

Hydrogen permeability was calculated according to the following expression [19]:

(3) $$j=\Phi \times \frac{d}{\Delta P^{0.5}},$$

where *d*—sample thickness, Δ*P*—pressure difference.

### 2.4. Ab Initio Calculations

The binding energy for a hydrogen atom in Nb-Ni-Ti-Zr-Co high entropy alloys was theoretically investigated using first principles. All calculations were performed within the framework of the density functional theory using the optimized norm-preserving Vanderbilt pseudopotential [20], realized in the ABINIT software package [21,22] within virtual crystal approximation (VCA) [23,24]. To describe the exchange and correlation effects, the generalized gradient approximation in the form of Perdew, Burke, and Ernzerhof was used [25]. The lattices were completely relaxed. The relaxation was considered complete when the forces acting on the atoms were less than 25 meV/Å. To carry out the structural optimization and relaxation of the Nb-Ni-Ti-Zr-Co system for discussion, a cell with 2 atoms with mixing potentials and 1 H atom was adopted, and the k meshes were chosen to be 10 × 10 × 10 for BCC structures. The cutoff energy for the plane-wave basis was set at 820 eV.

The binding energy of hydrogen atom in HEAs was calculated according to the following expression:

*E_b_* = *E*(A_2_) + ½*E*(H_2_) − *E*(A_2_H), (4)

where *E*(A_2_) is the energy of pure alloy in the presence of two mixing atoms in the cell; *E*(H_2_) is the energy of the hydrogen molecule; *E*(A_2_H) is the energy of the structure when there is a hydrogen atom in it.

## 3. Results

### 3.1. Microstructure and Phase Composition

**Figure 2:**
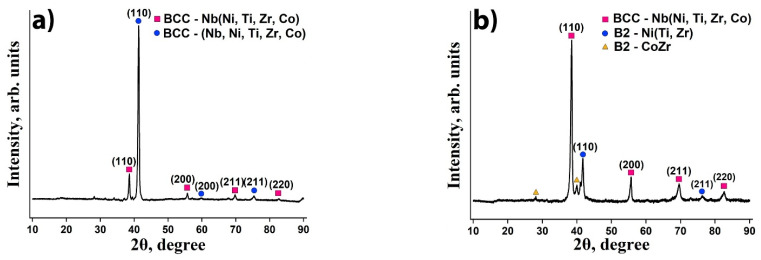
XRD patterns of Nb_20_Ni_20_Ti_20_Zr_20_Co_20_ (**a**) and Nb_40_Ni_25_Ti_18_Zr_12_Co_5_ (**b**) high entropy alloys annealed at 800 °C for 10 h.

The XRD patterns of Nb_20_Ni_20_Ti_20_Zr_20_Co_20_ and Nb_40_Ni_25_Ti_18_Zr_12_Co_5_ high entropy alloys after annealing at 800 °C for 10 h are presented in Figure 2. According to XRD measurements, equimolar Nb_20_Ni_20_Ti_20_Zr_20_Co_20_ HEA consists of two BCC phases with lattice constants of 3.306 Å and 3.093 Å (Figure 2a). For non-equimolar Nb_40_Ni_25_Ti_18_Zr_12_Co_5_ alloy, an Nb-rich BCC phase with the lattice constant of 3.306 Å and a B2-Ni(Ti, Zr) phase with the lattice constant of 3.060 Å were observed (Figure 2b). Additionally, weakly pronounced peaks of B2-CoZr compound were detected for the non-equimolar alloy.

**Figure 3:**
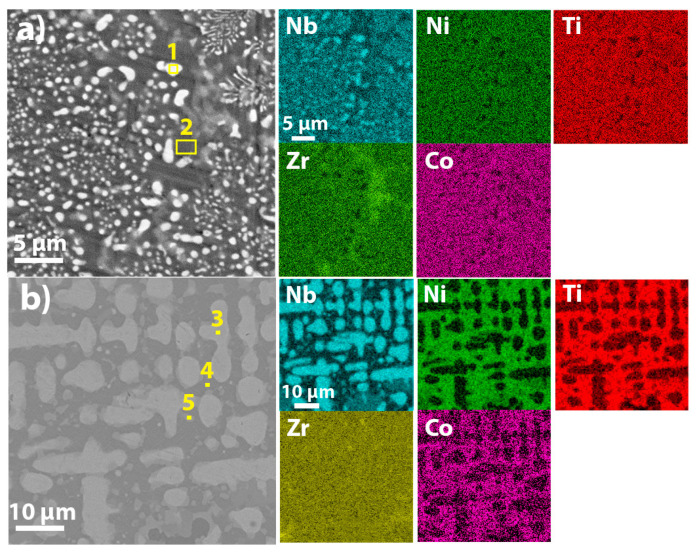
SEM images of Nb_20_Ni_20_Ti_20_Zr_20_Co_20_ (**a**) and Nb_40_Ni_25_Ti_18_Zr_12_Co_5_ (**b**) HEAs and corresponding EDS maps.

Figure 3a shows the SEM image and corresponding EDS maps of the equimolar Nb_20_Ni_20_Ti_20_Zr_20_Co_20_ alloy. Two major regions with different contrast can be seen: a light-grey contrasted Nb-rich BCC phase (region 1) with a globular and dendritic structure and a dark-grey contrasted—(Nb, Ni, Ti, Zr, Co) BCC phase (region 2), which has elemental content close to equimolar composition (Table 1).

The microstructure of non-equimolar Nb_40_Ni_25_Ti_18_Zr_12_Co_5_ (Figure 3b) high entropy alloy is represented by a light-grey contrasted Nb-rich BCC phase with dendritic structure (region 3) embedded in dark-grey contrasted matrix of B2-Ni(Ti, Zr) and B2-CoZr phases (region 4). Similar morphology and phases were observed by Tang et al. for Nb_40_Ti_18_Zr_12_Ni_30_ alloy [15]. Additionally, light-grey globular inclusions with high concentration of Nb (31 at.%) were detected in region 5. Since this also has high Ni content, it is assumed that this region corresponds to an Nb-enriched BCC-phase surrounded by a B2-Ni(Ti, Zr) phase.

### 3.2. Hydrogen Permeability

**Figure 4:**
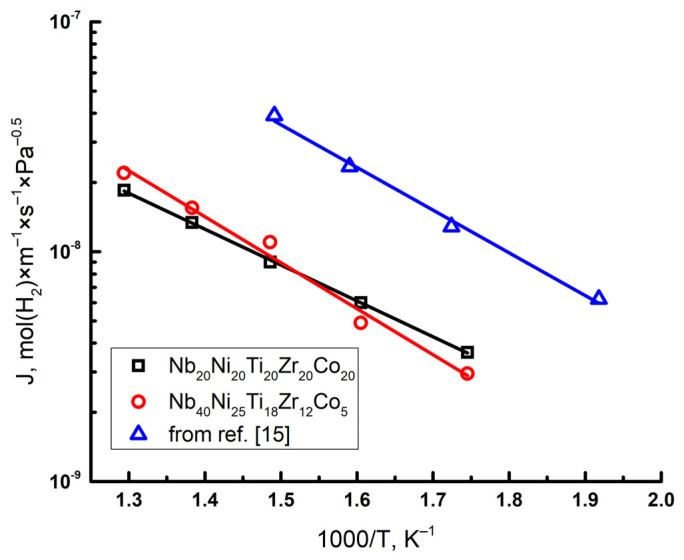
Hydrogen permeability of HEAs depending on temperature.

Figure 4 shows permeability curves for the HEA samples. The permeability of Nb_40_Ni_25_Ti_18_Zr_12_Co_5_ obtained by Tang et al. was added to Figure 4 for comparison. To analyze the temperature effect, Arrhenius plots were constructed based on the data of linear approximation of the permeability rate on inverse temperature. The permeability of the equimolar alloy is higher at lower temperatures (300–350 °C), but lower at higher temperatures compared to the non-equimolar alloy. Approximation of the permeability by the Arrhenius equation for HEA alloys showed that the activation energy of permeability for Nb_20_Ni_20_Ti_20_Co_20_Zr_20_ is 30 kJ/mol with pre-exponential factor 1.8×10^−6^, and for Nb_40_Ni_25_Ti_18_Zr_12_Co_5_ the activation energy is 38 kJ/mol with pre-exponential factor 1×10^−5^. It can be seen that the activation energy of the equimolar high-entropy alloy is significantly lower than that for the non-equimolar Nb_40_Ti_18_Zr_12_ Ni_25_Co_5_ alloy. According to permeability tests, it was revealed that equimolar HEA is less susceptible to hydrogen embrittlement. The Nb_40_Ni_25_Ti_18_Zr_12_Co_5_ alloy failed (crashed) after the 12 h permeation experiment. Under the same conditions, the equimolar HEA worked for more than 26 h without loss of performance.

### 3.3. Lattice Constants and Hydrogen Binding Energy in HEAs

Table 2 summarizes the ab initio calculation results for lattice parameters and hydrogen binding energies in different phases of the HEAs. The overall dependence of the lattice constant changes depending on the elemental composition is in good correlation with the experimental results. The best agreement between the calculated results and experimental lattice parameters was observed for high niobium concentrations in the BCC lattice. However, in all cases the calculated lattice constant is underestimated, which can be caused by temperature effect, lattice strains in as-fabricated HEAs and features of the VCA.

The difference in the changes in lattice parameters depending on the location of hydrogen atoms in BCC structure was found. The hydrogen atom in octahedral (O) sites leads to a lattice distortion along *c* axis at the octahedral coordination and all lattice expansion at the tetrahedral coordination in both BCC-Nb(Ni,Ti,Zr,Co) and BCC-(Nb,Ni,Ti,Zr,Co) phases.

The highest binding energy for hydrogen atoms was found at the high Nb concentration BCC lattice, which probably implies higher absorption of the hydrogen in regions with BCC-Nb(Ni,Ti,Zr,Co) phase. The decrease in Nb concentration leads to the decrease in the hydrogen binding energy in both T and O sites. In the Nb_15_Ni_20_Ti_16_Zr_29_Co_19_ and Nb_7.7_Ni_41.6_Ti_26.32_Zr_16.6_Co_7.59_ systems, the location of the hydrogen atom in tetrahedral sites leads to system instability—a hydrogen atom leaves these sites. The lowest binding energy of the hydrogen atoms in the octahedral sites and the unstable tetrahedral configuration of hydrogen atoms can lead to the high hydrogen diffusivity in the BCC-(Nb,Ni,Ti,Zr,Co) lattice compared to Nb-rich BCC phase.

## 4. Discussion

The synthesized equimolar and non-equimolar HEAs have high hydrogen permeability, which depends on their microstructure and phase composition. The equimolar alloy has lower activation energy, and correspondingly higher permeability at lower temperatures (300–350 °C). This alloy is also more resistant to hydrogen embrittlement than the non-equimolar one containing a higher concentration of niobium. In the case of equimolar Nb_20_Ni_20_Ti_20_Zr_20_Co_20_ HEA, a fine-dispersed structure with Nb-rich BCC-Nb(Ni,Ti,Zr,Co) and BCC-(Nb,Ni,Ti,Zr,Co) phases was obtained. Several studies have observed higher permeability of the multicomponent alloys with fine-dispersed structure, for example, for Nb–Ti–Co and Nb–Ni–Ti alloys [26,27,28]. Hashi et al. [26] and Yan et al. [27] also observed high resistance to hydrogen embrittlement for the fine grained alloy compared to coarse grained. Thus, the homogeneous fine microstructure of Nb_20_Ni_20_Ti_20_Zr_20_Co_20_ HEA can have a positive effect both on the permeability of the alloy due to the large number of phase boundaries and on hydrogen embrittlement resistance. It is known that permeability depends on many factors, but the most important are solubility and diffusivity within the crystal lattice [29]. Niobium and its alloys are characterized by high hydrogen permeability due to high solubility limits and the relatively high diffusion mobility of hydrogen [30]. Based on ab initio calculations, hydrogen prefers to occupy tetrahedral sites in the Nb-based BCC lattice, which also agrees well with the results of other works [31]. The Nb-based crystal lattice also has a larger volume due to the larger lattice parameter of 3.306 A for the Nb-based BCC-Nb(Ni, Ti, Zr, Co) compared to the equimolar BCC-(Nb, Ni, Ti, Zr, Co) with the lattice parameter of 3.06 A. This provides higher solubility of the Nb-based BCC lattice in the HEAs. In turn, the low binding energy of hydrogen in the interstitial sites of the equimolar alloy can provide better diffusion mobility of hydrogen in the lattice but lower hydrogen absorption and hydrogen embrittlement. We believe that the competition between solubility and diffusivity can explain the observed difference in the permeability of HEAs. Based on the XRD data, the main phase in the equimolar alloy is BCC-(Nb, Ni, Ti, Zr, Co), while in the non-equimolar is BCC-Nb(Ni, Ti, Co). It should also be noted that the presence of secondary phases B2-Ni(Ti, Zr) and B2-CoZr in the Nb_40_Ni_25_Ti_18_Zr_12_Co_5_ alloy can negatively affect its hydrogen permeability [26]. Thus, it is assumed that the high permeability of the Nb_40_Ni_25_Ti_18_Zr_12_Co_5_ alloy at higher temperatures (400–500 °C) is provided mainly by the more permeable Nb-based BCC lattice with higher hydrogen solubility. While for the equimolar alloy, the high diffusivity of hydrogen through the BCC-(Nb, Ni, Ti, Zr, Co) along with the fine-dispersed structure provide lower activation energy and higher permeability at lower temperatures. In addition, it should be noted that the distortions of the crystal lattice in HEAs due to the difference in the atomic sizes in the structure make an additional contribution to the increase in hydrogen solubility. This can cause the difference in the behavior of permeability depending on the temperature for pure niobium and Nb-based multicomponent BCC structures.

## 5. Conclusions

High-entropy alloys with equimolar and non-equimolar compositions were synthesized by arc melting. Their microstructure, phase composition, hydrogen binding energy in interstitial sites as well as hydrogen permeability at 300–500 °C were studied. The following conclusions was made:

1. A fine-grained microstructure with BCC-(Nb, Ni, Ti, Zr, Co) and BCC-Nb(Ni, Ti, Zr, Co) lattices is formed in the equimolar Nb_20_Ni_20_Ti_20_Zr_20_Co_20_ alloy, while coarser dendritic microstructure with Nb-enriched BCC-Nb(Ni, Ti, Co), B2-Ni(Ti, Zr) and B2-CoZr phases is presented in the Nb_40_Ni_25_Ti_18_Zr_12_Co_5_ alloy.
2. The lattice parameter and hydrogen binding energy in the interstitial sites are higher for the Nb-enriched BCC lattice than that for the BCC lattice with near equimolar composition, which indicates higher hydrogen solubility in the Nb-enriched phase. The hydrogen binding energy decreases in both tetrahedral and octahedral sites in the BCC-(Nb, Ni, Ti, Zr, Co) lattice, which apparently causes higher diffusivity of hydrogen.
3. The Nb_20_Ni_20_Ti_20_Zr_20_Co_20_ alloy shows lower activation energy and higher permeability at temperatures of 300–350 °C as well as higher resistance to hydrogen embrittlement.

The obtained results give prospects for the development of HEA-based metallic membranes for hydrogen separation and purification.

## Figures and Tables

**Table 1:** Elemental concentration in different regions as depicted in Figure 3.

Sample	Region	Concentration, at. %					Possible Phases
		Nb	Ni	Ti	Zr	Co	
Nb_20_Ni_20_Ti_20_Zr_20_Co_20_	1	74	6	9	5	6	BCC-Nb(Ni, Ti, Zr, Co)
	2	15	20	16	29	19	BCC-(Nb, Ni, Ti, Zr, Co)
Nb_40_Ni_25_Ti_18_Zr_12_Co_5_	3	88.8	3.7	6.5	-	0.9	BCC-Nb(Ni, Ti, Co)
	4	7.7	41.6	26.3	16.6	7.6	B2-Ni(Ti, Zr), B2-CoZr
	5	31	36	17.7	8.5	6.5	BCC-Nb(Ni, Ti, Zr, Co), B2-Ni(Ti, Zr)

**Table 2:** Lattice parameters and binding energies, *Eb*, for the hydrogen atom.

Alloy	Phase	Lattice Constant, Å				*E_b_*, eV
		Exp	*a*, Calc	*b*, Calc	*c*, Calc	
Nb_74_Ni_6_Ti_9_Zr_5_Co_6_	pure	3.306	3.192	3.192	3.192	-
	H in T sites		3.275	3.293	3.293	0.760
	H in O sites		3.137	3.137	3.586	0.441
Nb_15_Ni_20_Ti_16_Zr_29_Co_19_	pure	3.090	2.979	2.979	2.979	-
	H in O sites		2.727	2.727	3.863	0.248
Nb_88.8_Ni_3.7_Ti_6.5_Co_0.9_	pure	3.306	3.251	3.251	3.251	-
	H in T sites		3.331	3.354	3.354	1.037
	H in O sites		3.196	3.196	3.641	0.777
Nb_7.7_Ni_41.6_Ti_26.32_Zr_16.6_Co_7.59_	pure	3.060	2.855	2.855	2.855	-
	H in O sites		2.609	2.609	3.717	0.686

## Data Availability

Data are contained within the article.
